# Non-contiguous finished genome sequence and description of *Megasphaera massiliensis* sp. nov.

**DOI:** 10.4056/sigs.4077819

**Published:** 2013-08-07

**Authors:** Roshan Padmanabhan, Jean-Christophe Lagier, Nicole Prisca Makaya Dangui, Caroline Michelle, Carine Couderc, Didier Raoult, Pierre-Edouard Fournier

**Affiliations:** 1 Aix-Marseille Université, URMITE, Faculté de médecine, Marseille, France

**Keywords:** *Megasphaera massiliensis*, genome, culturomics, taxonogenomics

## Abstract

*Megasphaera massiliensis* strain NP3^T^ sp. nov. is the type strain of *Megasphaera massiliensis* sp. nov., a new species within the genus *Megasphaera*. This strain, whose genome is described here, was isolated from the fecal flora of an HIV-infected patient. *M. massiliensis* is a Gram-negative, obligate anaerobic coccobacillus. Here we describe the features of this organism, together with the complete genome sequence and annotation. The 2,661,757 bp long genome (1 chromosome but no plasmid) contains 2,577 protein-coding and 61 RNA genes, including 5 rRNA genes.

## Introduction

*Megasphaera massiliensis* sp. nov. strain NP3^T^ (= CSUR P245 = DSM 26228) is the type strain of *M. massiliensis* sp. nov. This bacterium is a Gram-negative, non-sporulating, anaerobic and non-motile coccobacillus that was isolated from the stool of an HIV-infected patient as part of a culturomics study designed to cultivate individually all bacterial species within human feces [1,2].

The current classification of prokaryotes is based on a combination of phenotypic and genotypic characteristics [3,4] including 16S rRNA gene phylogeny, G + C content and DNA–DNA hybridization (DDH). Despite being considered as a “gold standard”, these tools exhibit several drawbacks [5,6]. To date, almost 4,000 bacterial genomes have been sequenced [7] and the cost of genomic sequencing is constantly decreasing. Therefore, we recently proposed the addition of genomic information to phenotypic criteria, including the protein profile, for the description of new bacterial species [8-29].

The genus *Megasphaera* (Rogosa 1971), created in 1971 [30], currently contains 5 species including *M. cerevisiae* (Engelmann and Weiss 1986) [31], *M. elsdenii* (Gutierrez *et al.* 1959) [30], *M. micronuciformis* (Marchandin *et al.* 2003) [32], *M. paucivorans* (Juvonen and Suihko 2006) [33] and *M. sueciensis* (Juvonen and Suihko 2006) [33]. The type species, *M. elsdenii* (Gutierrez *et al.* 1959) [30], originally classified in the *Peptostreptococcus* genus (Gutierrez *et al.* 1959), was later reclassified within a new genus, *Megasphaera* (Rogosa 1971), in the family *Veillonellaceae* (Rogosa 1971) [30]. It is an obligately anaerobic, lactate-fermenting, gastrointestinal microbe of ruminant and non-ruminant mammals, including humans. It was also isolated in a case of human endocarditis [34]. The genome from *M. elsdenii* strain DSM 20460, isolated from the rumen of sheep, was recently sequenced [35]. *M. cerevisiae* [31], *M. micronuciformis* [32], *M. paucivorans* and *M. sueciensis* [33] are brewery-associated species. Here we present a summary classification and a set of features for *M. massiliensis* sp. nov. strain NP3^T^ (= CSUR P245 = DSM 26228) together with the description of the complete genome sequencing and annotation. These characteristics support the circumscription of the species *M. massiliensis*.

## Classification and features

A stool sample was collected from a 32-year-old HIV-infected patient living in Marseille, France. The patient gave written informed consent for the study. The study was approved by the Ethics Committee of the Institut Fédératif de Recherche IFR48, Faculty of Medicine, Marseille, France, under agreement number 09-022.

**Figure 1:**
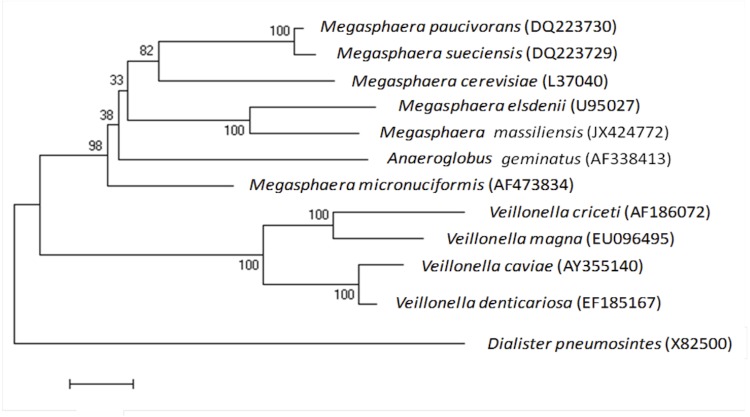
Phylogenetic tree highlighting the position of *Megasphaera massiliensis* strain NP3^T^ relative to other type strains within the genus *Megasphaera* and other members of the family *Veillonellaceae*. GenBank accession numbers are indicated in parentheses. Sequences were aligned using CLUSTALW, and phylogenetic inferences obtained using the maximum-likelihood method within the MEGA software. Numbers at the nodes are percentages of bootstrap values obtained by repeating the analysis 500 times to generate a majority consensus tree. *Dialister pneumosintes* was used as outgroup. The scale bar indicates a 1% nucleotide sequence divergence.

The fecal specimen was preserved at -80°C after collection. Strain NP3^T^ (Table 1) was isolated in January 2012 by cultivation on 5% sheep blood agar in anaerobic condition at 37°C, following a 7-day preincubation of the stool specimen in an anaerobic blood culture bottle enriched with sterile 5% sheep rumen fluid and 5% sheep blood. The strain exhibited a nucleotide sequence similarity with other members of the genus *Megasphaera* ranging from 91.5% with *M. cerevisiae* strain PAT1^T^ to 95.8% with *M. elsdenii* strain ATCC 25940^T^, its closest validated phylogenetic neighbor (Figure 1). These values were lower than the 98.7% 16S rRNA gene sequence threshold recommended by Stackebrandt and Ebers to delineate a new species without carrying out DNA-DNA hybridization [4].

**Table 1:** Classification and general features of *Megasphaera massiliensis* strain NP3^T^ according to the MIGS recommendations [36]

**MIGS ID**	**Property**	**Term**	**Evidence code^a^**
	Current classification	Domain *Bacteria*	TAS [37]
		Phylum *Firmicutes*	TAS [38-40]
		Class *Negativicutes*	TAS [41]
		Order *Selenomonadales*	TAS [41]
		Family *Veillonellaceae*	TAS [41-43]
		Genus *Megasphaera*	TAS [30,32,43,44]
		Species *Megasphaera massiliensis*	IDA
		Type strain NP3^T^	IDA
	Gram stain	Negative	IDA
	Cell shape	coccobacilli	IDA
	Motility	non motile	IDA
	Sporulation	nonsporulating	IDA
	Temperature range	mesophilic	IDA
	Optimum temperature	37°C	IDA
MIGS-6.3	Salinity	unknown	
MIGS-22	Oxygen requirement	anaerobic	IDA
	Carbon source	unknown	
	Energy source	unknown	
MIGS-6	Habitat	human gut	IDA
MIGS-15	Biotic relationship	free living	IDA
MIGS-14	Pathogenicity Biosafety level Isolation	unknown 2 human feces	
MIGS-4	Geographic location	France	IDA
MIGS-5	Sample collection time	January 2012	IDA
MIGS-4.1	Latitude	43.296482	IDA
MIGS-4.1	Longitude	5.36978	IDA
MIGS-4.3	Depth	Surface	IDA
MIGS-4.4	Altitude	0 m above sea level	IDA

Evidence codes - IDA: Inferred from Direct Assay; TAS: Traceable Author Statement (i.e., a direct report exists in the literature); NAS: Non-traceable Author Statement (i.e., not directly observed for the living, isolated sample, but based on a generally accepted property for the species, or anecdotal evidence). These evidence codes are from the Gene Ontology project [45]. If the evidence is IDA, then the property was directly observed for a live isolate by one of the authors or an expert mentioned in the acknowledgements.

**Figure 2:**
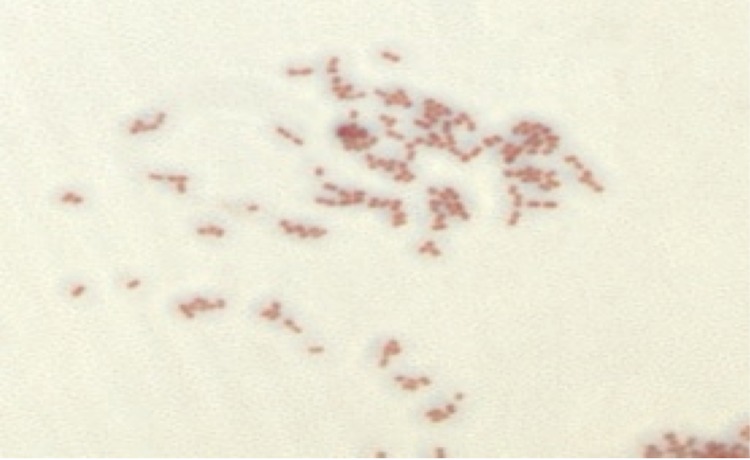
Gram staining of *Megasphaera massiliensis* strain NP3^T^

**Figure 3:**
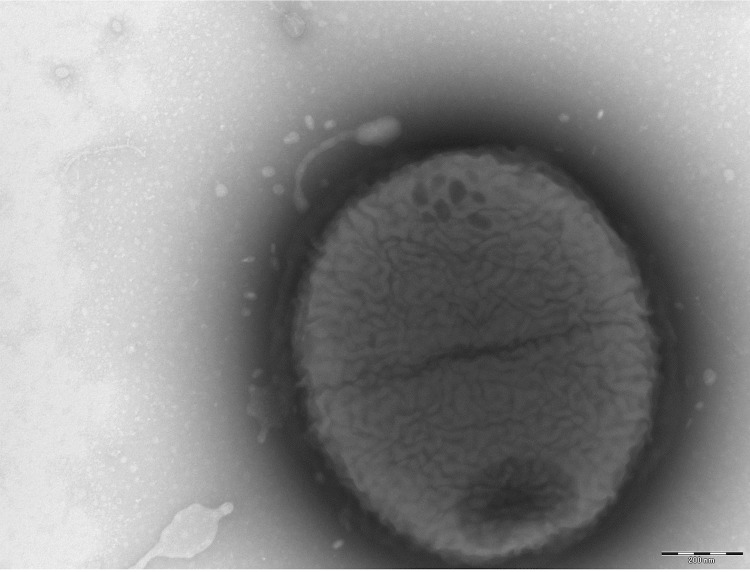
Transmission electron microscopy of *M. massiliensis* strain NP3^T^, using a Morgani 268D (Philips) at an operating voltage of 60kV. The scale bar represents 200 nm.

Different growth temperatures (25, 30, 37, 45°C) were tested. Growth occurred between 30 and 45°C, and optimal growth was observed at 37°C. Colonies were transparent and smooth with a diameter of 0.5 to 1 mm on blood-enriched Columbia agar (BioMérieux). Growth of the strain was tested in 5% sheep blood agar (BioMérieux) under anaerobic and microaerophilic conditions using GENbag anaer and GENbag microaer systems, respectively (BioMérieux), and in the presence of air, with or without 5% CO_2_. Growth only occurred in anaerobic atmosphere. No growth was observed under aerobic conditions and microaerophilic conditions. A motility test was negative. Cells grown on agar are Gram-negative coccobacilli (Figure 2), with a mean diameter of 0.87 µm and the presence of phages in electron microscopy (Figure 3).

Strain NP3^T^ exhibited oxidase, but no catalase activity. Using RAPID 32A identification strips (BioMérieux), positive reactions were observed for α-glucosidase and β-glucosidase. Negative reactions were observed for urease, arginine dihydrolase, α and β-galactosidase, β-galactosidase-6-phosphate, α-arabinosidase, β-glucuronidase, N-acetyl-β-glucosanimidase, mannose and raffinose fermentation, α-fucosidase, alkanine phosphatase, arginine arylamidase, proline arylamidase, leucyl glycine arylamidase, phenylalanine arylamidase, leucine arylamidase, pyroglutamic acid arylamidase, tyrosine arylamidase, alanine arylamidase, glycine arylamidase, histidine arylamidase, glutamyl glutamic acid arylamidase and serine arylamidase. Carbohydrate metabolism was examined using an API 50CH strip (BioMerieux). Positive reactions were observed for potassium gluconate, potassium 5-cetogluconate, aesculin, salicine, N-acetylglucosamine, and arbutine production, and L-arabinose, D-ribose, D-xylose, D-galactose, D-glucose, D-fructose, D-mannose, L-rhamnose, D-mannitol, D-sorbitol, D-celiobiose, D-maltose, D-lactose, D-trehalose, gentiobiose, L-fucose and D-arabitol fermentation. Weak reactions were observed for amygdaline and potassium 2-cetogluconate production, and glycerol and D-arabinose fermentation. Table 2 summarizes the differential phenotypic characteristics of *M. massiliensis*, *M. elsdenii* and *M. micronuciformis*. *M. massiliensis* strain NP3^T^ was susceptible to amoxicillin, amoxicillin-clavulanic acid, ceftriaxone, imipenem and doxycycline but resistant to vancomycin, erythromycin, rifampicin, trimethoprim-sulfamethoxazole, metronidazole and ciprofloxacin.

**Table 2:** Differential characteristics of *M. massiliensis* strain NP3^T^, *M. elsdenii* strain DSM 20460 and *M. micronuciformis* strain AIP 412-00^T^.^†^

**Properties**	***M. massiliensis***	***M. elsdenii***	***M. micromuciniformis***
Cell diameter (µm)	0.87	1.5-3.0	0.4-0.6
Oxygen requirement	anaerobic	anaerobic	Anaerobic
Pigment production	+	+	–
Gram stain	–	–	–
Motility	–	–	–
Endospore formation	–	–	–
Indole production	–	na	–
**Production of**			
Catalase	–	–	–
Oxidase	+	+	na
Nitrate reductase	na	–	–
Urease	–	–	na
β-galactosidase	–	–	na
N-acetyl-glucosamine	na	–	na
**Acid production from**			
Arabinose	w	–	–
Ribose	+	–	na
Mannose	–	–	–
Mannitol	+	+	–
Raffinose	–	–	–
Sucrose	–	–	–
Glycerol	w	–	–
Sorbitol	+	–	na
Arabitol	+	–	na
Galactose	+	+	–
D-glucose	+	+	–
D-fructose	+	+	–
D-maltose	+	+	–
D-lactose	+	+	–
**Hydrolysis of gelatin**	+	+	–
Habitat	Human gut	Sheep rumen	Liver abscess, whitlow

na = data not available; w = weak

**Figure 4:**
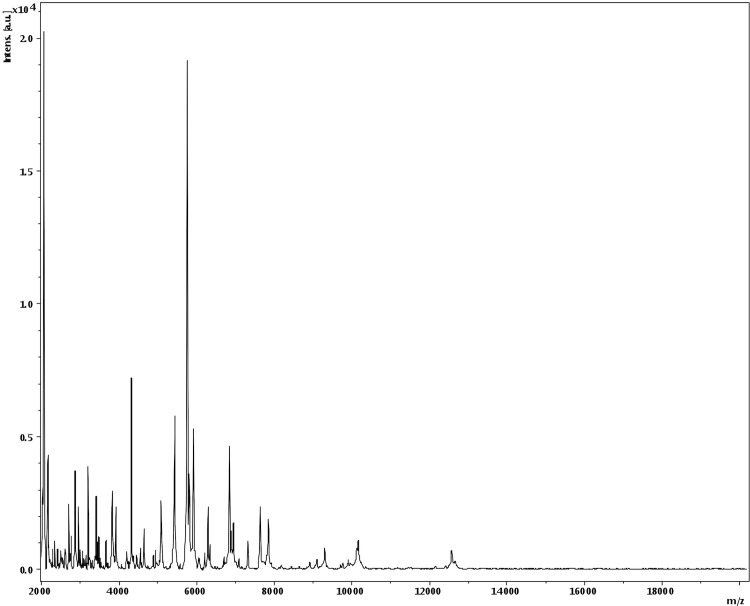
Reference mass spectrum from *M. massiliensis* strain NP3^T^. Spectra from 12 individual colonies were compared and a reference spectrum was generated.

**Figure 5:**
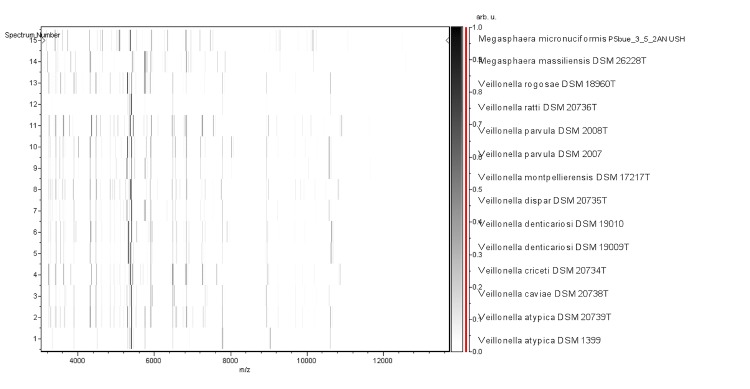
Gel view comparing the *M. massiliensis* NP3^T^ spectrum with those of *M. micronuciformis* and *Veillonella* species. The Gel View displays the raw spectra of all loaded spectrum files arranged in a pseudo-gel like look. The x-axis records the m/z value. The left y-axis displays the running spectrum number originating from subsequent spectra loading. The peak intensity is expressed by a Gray scale scheme code. The color bar and the right y-axis indicate the relation between the color a peak is displayed and the peak intensity in arbitrary units.

Matrix-assisted laser-desorption/ionization time-of-flight (MALDI-TOF) MS protein analysis was carried out as previously described [46] using a Microflex spectrometer (Brüker Daltonics, Germany). Briefly, a pipette tip was used to pick one isolated bacterial colony from a culture agar plate and spread it as a thin film on a MTP 384 MALDI-TOF target plate (Bruker Daltonics). Twelve distinct deposits were done for strain NP3^T^ from 12 isolated colonies. Each smear was overlaid with 2 µL of matrix solution (a saturated solution of alpha-cyano-4-hydroxycinnamic acid) in 50% acetonitrile, 2.5% tri-fluoracetic acid and allowed to dry for five minutes. Spectra were recorded in the positive linear mode for the mass range from 2,000 to 20,000 Da (parameter settings: ion source 1 (ISI), 20kV; IS2, 18.5 kV; lens, 7 kV). A spectrum was obtained after 675 shots with variable laser power. The time of acquisition was between 30 seconds and 1 minute per spot. The 12 NP3^T^ spectra were imported into the MALDI Bio Typer software (version 2.0, Bruker) and analyzed by standard pattern matching (with default parameter settings) against the main spectra of 3,769 bacteria, including the spectra from *M. micronuciformis*, *Veillonella atypica*, *V. caviae*, *V. criceti*, *V. denticariosi*, *V. dispar*, *V. montpellierensis*, *V. parvula*, *V. ratti* and *V. rogosae*, that were used as reference data (Figures 4 and 5). The method of identification included the m/z from 3,000 to 15,000 Da. For every spectrum, 100 peaks at most were taken into account and compared with the spectra in the database. The MALDI-TOF score enabled the predictive identification and discrimination of the tested species from those in a database: a score > 2 with a validated species enabled identification at the species level, and a score < 1.7 did not enable any identification. No significant score was obtained for strain NP3^T^ against the Brüker database, suggesting that our isolate was not a member of a known species. We added the spectrum from strain NP3^T^ to our database for future reference (Figure 4). Figure 5 shows the MALDI-TOF MS spectrum differences between *M. massiliensis* and other *Megasphaera* and *Veillonella* species (Figure 5).

## Genome sequencing information

### Genome project history

The organism was selected for sequencing on the basis of its phenotypic differences, phylogenetic position and 16S rRNA similarity to other members of the genus *Megasphaera*, and is part of a study of the human digestive flora aiming at isolating all bacterial species within human feces [1,2]. It was the third genome of a *Megasphaera* species and the first sequenced genome of *M. massiliensis* sp. nov. The GenBank ID is CAVO00000000 and consists of 106 large contigs. Table 3 shows the project information and its association with MIGS version 2.0 compliance [47].

**Table 3:** Project information

**MIGS ID**	**Property**	**Term**
MIGS-31	Finishing quality	High-quality draft
MIGS-28	Libraries used	Paired-end 3kb library
MIGS-29	Sequencing platforms	454 GS FLX Titanium
MIGS-31.2	Fold coverage	19 ×
MIGS-30	Assemblers	Newbler version 2.5.3
MIGS-32	Gene calling method	Prodigal
	INSDC ID	PRJEB645
	Genbank ID	CAVO00000000
	Genbank Date of Release	June 4, 2013
	Project relevance	Study of the human gut microbiome

### Growth conditions and DNA isolation

*Megasphaera massiliensis* strain NP3^T^ sp. nov. (= CSUR P245 = DSM 26228) was grown anaerobically on 5% sheep blood-enriched agar (BioMérieux) at 37°C. Ten petri dishes were spread and resuspended in 3 × 100µl of G2 buffer (EZ1 DNA Tissue kit, Qiagen). A first mechanical lysis was performed using glass powder on the Fastprep-24 device (MP Biomedicals, Ilkirch, France) during 2 × 20 seconds. DNA was then treated with 2.5 µg/µL lysozyme treatment (30 minutes at 37°C) and extracted using a BioRobot EZ 1 Advanced XL (Qiagen). The DNA was then concentrated and purified using a Qiamp kit (Qiagen). The yield and the concentration were measured using the Quant-it Picogreen kit (Invitrogen) on the Genios_Tecan fluorometer at 82.2 ng/µl.

### Genome sequencing and assembly

A paired-end sequencing strategy was used (Roche). The library was pyrosequenced on a GS FLX Titanium sequencere (Roche). This project was loaded on a 1/4 region on PTP Picotiterplate (Roche). Five µg of DNA were mechanically fragmented on the Covaris device (KBioScience-LGC Genomics, Teddington, UK) using miniTUBE-Red 5Kb. The DNA fragmentation was visualized through the Agilent 2100 BioAnalyzer on a DNA labchip 7500 with an optimal size of 3.3 kb. After PCR amplification through 17 cycles followed by double size selection, the single stranded paired-end library was then loaded on a DNA labchip RNA pico 6000 on the BioAnalyzer. The pattern showed an optimum at 613 bp and the concentration was quantified on a Genios Tecan fluorometer at 3.48 pg/µL. The library concentration equivalence was calculated at 5.21E+09 molecules/µL. The library was stored at -20°C until further use, and the library was clonally amplified with 0.5 cpb in 3 emPCR reactions with the GS Titanium SV emPCR Kit (Lib-L) v2 (Roche). The yield of the emPCR was 9.99%, in the range of 5 to 20% from the Roche procedure. Approximately 790,000 beads were loaded on the GS Titanium PicoTiterPlates PTP Kit 70x75 and sequenced with the GS FLX Titanium Sequencing Kit XLR70 (Roche). The run was performed overnight and then analyzed on the cluster through the gsRunBrowser and Newbler Assembler (Roche). A total of 186,153 passed filter wells generated 61.97 Mb with a length average of 332 bp. The filter-passed sequences were assembled using Newbler with 90% identity and 40 bp as overlap. The final assembly identified 114 large contigs (>1,500 bp) arranged into 28 scaffolds and generated a genome size of 2.66 Mb, which corresponds to a coverage of 23.3× genome equivalent.

### Genome annotation

Prodigal [48] with default parameters was used to predict the Open Reading Frames (ORFs). The predicted ORFs were excluded if they spanned a sequencing gap region. Protein functional assessment was obtained by comparison with sequences in the GenBank [49] and Clusters of Orthologs Groups (COG) databases using BLASTP. The rRNA and tRNA were identified using RNAmmer [50] and tRNAscan-SE 1.21 [51] respectively. SignalP [52] and TMHMM [53] were used to predict signal peptides and transmembrane helices, respectively. ORFans were identified if their BLASTP E-value was lower than 1e-03 for alignment length greater than 80 amino acids. If alignment lengths were smaller than 80 amino acids, we used an E-value of 1e-05. Such parameter thresholds have already been used in previous works to define ORFans. Artemis [54] was used for data management and DNA Plotter [55] was used for visualization of genomic features. PHAST was used to identify, annotate and graphically display prophage sequences within bacterial genomes or plasmids [56]. To estimate the mean level of nucleotide sequence similarity at the genome level between *M. massiliensis* and another 5 members of the family *Veillonellaceae,* orthologous proteins were detected using the Proteinortho software with the following parameters: e-value 1e-5, 30% percentage of identity, 50% coverage and algebraic connectivity of 50% [57], and genomes compared two by two. For each pair of genomes, we determined the mean percentage of nucleotide sequence identity among orthologous ORFs using BLASTn.

## Genome properties

**Figure 6:**
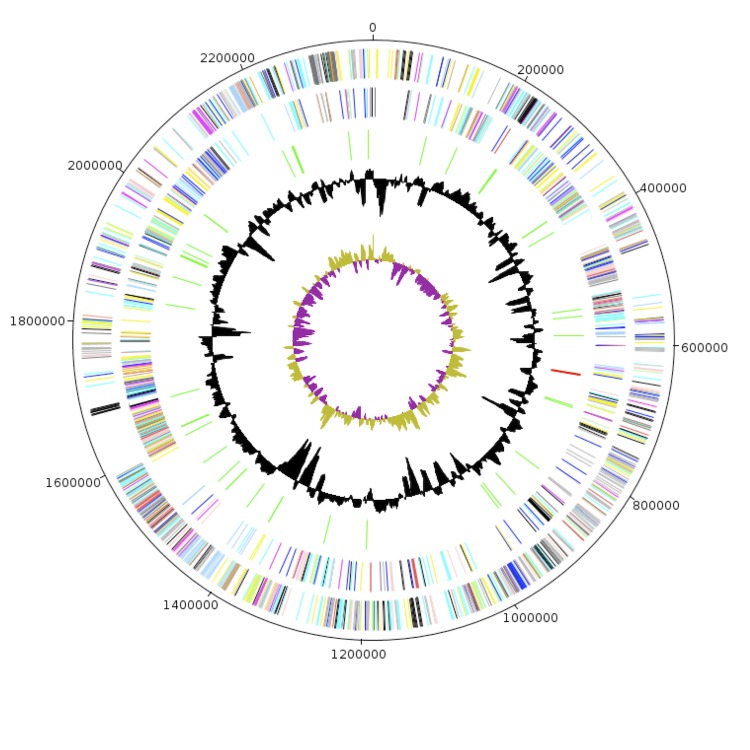
Graphical circular map of the *M. massiliensis* strain NP3^T^ chromosome. From the outside in: the outer two circles show open reading frames oriented in the forward and reverse (colored by COG categories) directions, respectively. The third circle displays the rRNA gene operon (red) and tRNA genes (green). The fourth circle shows the G+C% content plot. The inner-most circle shows the GC skew, purple and olive indicating negative and positive values, respectively.

The genome of *M. massiliensis* strain NP3^T^ is 2,661,757 bp long (in 28 scaffolds, 1 chromosome, and no plasmid) with a 50.2% GC content (Table 3 and Figure 6). Of the 2,577 predicted genes, 2,516 were protein-coding genes and there were 61 RNA genes. A total of 1,697 genes (65.8%) were assigned a putative function. A total of 248 genes (9.6%) were annotated as hypothetical proteins. The properties and the statistics of the genome are summarized in Tables 4 and 5. The distribution of genes into COGs functional categories is presented in Table 5.

**Table 4:** Nucleotide content and gene count levels of the genome

**Attribute**	Value	% of total^a^
Genome size (bp)	2,661,757	
DNA coding region (bp)	1,479,861	93.98
DNA G+C content (bp)	1,337,412	50.2
Coding region (bp)	1,479,861	93.98
Number of replicons	1	
Extrachromosomal elements	0	
Total genes	2,577	100
RNA genes	61	2.39
rRNA operons	2	
Protein-coding genes	2,516	97.63
Genes with function prediction	1,697	65.8
Genes assigned to COGs	1,892	73.41
Genes with peptide signals	60	2.38
Genes with transmembrane helices	530	21.0
CRISPR repeats	7	

^a^ The total is based on either the size of the genome in base pairs or the total number of protein coding genes in the annotated genome.

**Table 5:** Number of genes associated with the 25 general COG functional categories

**Code**	**Value**	**%age**^a^	**Description**
J	138	5.48	Translation
A	0	0	RNA processing and modification
K	120	4.77	Transcription
L	118	4.69	Replication, recombination and repair
B	0	0	Chromatin structure and dynamics
D	23	0.91	Cell cycle control, mitosis and meiosis
Y	0	0	Nuclear structure
V	28	1.11	Defense mechanisms
T	27	1.07	Signal transduction mechanisms
M	103	4.09	Cell wall/membrane biogenesis
N	0	0	Cell motility
Z	0	0	Cytoskeleton
W	0	0	Extracellular structures
U	24	0.95	Intracellular trafficking and secretion
O	52	2.07	Post-translational modification, protein turnover, chaperones
C	147	5.84	Energy production and conversion
G	118	4.69	Carbohydrate transport and metabolism
E	163	6.48	Amino acid transport and metabolism
F	52	2.07	Nucleotide transport and metabolism
H	87	3.46	Coenzyme transport and metabolism
I	46	1.83	Lipid transport and metabolism
P	79	3.14	Inorganic ion transport and metabolism
Q	14	0.56	Secondary metabolites biosynthesis, transport and catabolism
R	217	8.62	General function prediction only
S	141	5.60	Function unknown
-	195	7.75	Not in COGs

^a^ The total is based on the total number of protein coding genes in the annotated genome.

## C**omparison with the genomes from *M. elsdenii*, *Megasphaera* species, *Veillonella dispar*, *V. parvula* and *Anaeroglobus geminatus***

The draft genome of *M. massiliensis* strain NP3^T^ (2.66 Mb) has a larger size than that of *M. elsdenii* (2.47 Mb), *V. parvula* (2.13 Mb), *V. dispar* (2.12 Mb), *A. geminatus* (1.79 Mb) and *M. micronuciformis* (1.77 Mb) respectively. *M. massiliensis* has a lower G + C content (50.2%) than *M. elsdenii* (52.8%) but higher than *V. parvula*, *V. dispar*, *M. micronuciformis* and *A. geminatus* (38.6, 38.8, 46.8 and 48.7%, respectively). *M. massiliensis* (2,516) has more predicted protein-coding genes than *M. elsdenii*, *A. geminatus, V. dispar, V. parvula, and M. micronuciformis* (2,219, 2,148, 1,954, 1,844 and 1,774, respectively) (Table 6). In addition, *M. massiliensis* shared a mean genomic sequence similarity of 81.84, 69.44, 63.68, 62.92 and 70.27% with *M. elsdenii, M. micronuciformis, V. dispar, V. parvula* and *A. geminatus* respectively (Table 6).

**Table 6:** Orthologous gene comparison and average nucleotide identity of *M. massiliensis* with other compared genomes ^†^

Species (GenBank accession number)	*M. massiliensis*	*M. elsdenii*	*M. micronuciformis*	*V. dispar*	*V. parvula*	*A. geminatus*
*M. massiliensis* (CAVO00000000))	**2,516**	1,289	1,189	987	999	1,159
*M. elsdenii* (HE576794)	81.84	**2,219**	1,175	980	989	1,145
*M. micronuciformis* (AECS00000000)	69.44	69.01	**1,774**	933	939	1,167
*V. dispar* (ACIK00000000)	63.68	63.08	64.92	**1,954**	1,081	893
*V. parvula* (ADFU00000000)	62.92	62.01	64.43	67.62	**1,844**	899
*A. geminatus* (AGCJ00000000)	70.27	70.50	74.22	63.87	62.99	**2,148**

^†^Upper right, numbers of orthologous genes; lower left, mean nucleotide identities of orthologous genes. Bold numbers indicate the numbers of genes or each genome.

*M. massiliensis* harbors two intact bacteriophages. Based on PHAST results, phage 1 of *M. massiliensis* was most closely related to *Clostridium* phage phi CD119 whereas phage 2 was most similar to *Bacillus* phage BCJA1c.

## Conclusion

On the basis of phenotypic, phylogenetic and genomic analyses, we formally propose the creation of *Megasphaera massiliensis* sp. nov. that contains the strain NP3^T^. This bacterial strain has been found in Marseille, France.

### Description of *Megasphaera massiliensis* sp. nov.

*Megasphaera massiliensis* (mas.il.ien’sis. L. gen. fem. n. *massiliensis*, of Massilia, the Latin name of Marseille where was cultivated strain NP3^T^). It has been isolated from the feces of a 32-year-old HIV-infected French patient.

Colonies were smooth and transparent with 0.5 to 1 mm in diameter on blood-enriched Columbia agar. Optimal growth is only achieved anaerobically and grows between 30 and 45°C, with optimal growth observed at 37°C. The strain is a Gram-negative, non-endospore forming, non motile coccobacillus. Positive for α-glucosidase, β-glucosidase, potassium gluconate, potassium 5-cetogluconate, aesculin, salicine, N-acetylglucosamine, and arbutine production. Positive for L-arabinose, D-ribose, D-xylose, D-galactose, D-glucose, D-fructose, D-mannose, L-rhamnose, D-mannitol, D-sorbitol, D-celiobiose, D-maltose, D-lactose, D-trehalose, gentiobiose, L-fucose and D-arabitol fermentation. Negative for urease, arginine dihydrolase, α and β-galactosidase, β-galactosidase-6-phosphate, α-arabinosidase, β-glucuronidase, N-acetyl-β-glucosanimidase, mannose and raffinose fermentation, α-fucosidase, alkanine phosphatase, arginine arylamidase, proline arylamidase, leucyl glycine arylamidase, phenylalanine arylamidase, leucine arylamidase, pyroglutamic acid arylamidase, tyrosine arylamidase, alanine arylamidase, glycine arylamidase, histidine arylamidase, glutamyl glutamic acid arylamidase and serine arylamidase. Weak reactions observed for amygdaline and potassium 2-cetogluconate production, and glycerol and D-arabinose fermentation. Cells are susceptible to amoxicillin, amoxicillin-clavulanic acid, ceftriaxone, imipenem and doxycycline, but resistant to vancomycin, erythromycin, rifampicin, trimethoprim/sulfamethoxazole, metronidazole, and ciprofloxacin. The G+C content of the genome is 50.2%. The 16S rRNA and genome sequences are deposited in Genbank under accession numbers JX424772 and CAVO00000000, respectively. The type strain NP3^T^ (= CSUR P245 = DSM 26228) was isolated from the fecal flora of an HIV-infected patient in Marseille, France.
